# Obesity is associated with lower bacterial vaginosis prevalence in menopausal but not pre-menopausal women in a retrospective analysis of the Women’s Interagency HIV Study

**DOI:** 10.1371/journal.pone.0248136

**Published:** 2021-03-08

**Authors:** Elizabeth Daubert, Kathleen M. Weber, Audrey L. French, Dominika Seidman, Katherine Michel, Deborah Gustafson, Kerry Murphy, Christina A. Muzny, Maria Alcaide, Anandi Sheth, Adaora A. Adimora, Gregory T. Spear

**Affiliations:** 1 Cook County Health/Hektoen Institute of Medicine, Chicago, IL, United States of America; 2 Department of Medicine, Stroger Hospital of Cook County, Chicago, IL, United States of America; 3 School of Medicine, University of California, San Francisco, CA, United States of America; 4 Department of Infectious Diseases, School of Medicine, Georgetown University, Washington, D.C., United States of America; 5 Department of Neurology, State University of New York Downstate Health Sciences Medical Center, Brooklyn, New York, United States of America; 6 Department of Medicine, Montefiore at AECOM, Bronx, NY, United States of America; 7 Division of Infectious Diseases, University of Alabama at Birmingham, Birmingham, Alabama, United States of America; 8 Division of Infectious Diseases, University of Miami Miller School of Medicine, Miami, Florida, United States of America; 9 Division of Infectious Diseases, Department of Medicine, Emory University School of Medicine, Atlanta, GA, United States of America; 10 Division of Infectious Diseases, Department of Medicine, University of North Carolina at Chapel Hill, Chapel Hill, NC, United States of America; 11 Hektoen Institute of Medicine, Chicago, IL, United States of America; University of Mississippi Medical Center, UNITED STATES

## Abstract

The vaginal microbiota is known to impact women’s health, but the biological factors that influence the composition of the microbiota are not fully understood. We previously observed that levels of glycogen in the lumen of the vagina were higher in women that had a high body mass index (BMI). Vaginal glycogen is thought to impact the composition of the vaginal microbiota. We therefore sought to determine if BMI was associated having or not having bacterial vaginosis (BV), as determined by the Amsel criteria. We also hypothesized that increased blood glucose levels could lead to the previously-observed higher vaginal glycogen levels and therefore investigated if hemoglobin A1c levels were associated with BV. We analyzed data from the Women’s Interagency HIV Study using multiple multivariable (GEE) logistic regression models to assess the relationship between BMI, BV and blood glucose. Women with a BMI >30 kg/m^2^ (obese) had a lower rate (multivariable adjusted OR 0.87 (0.79–0.97), p = 0.009) of BV compared to the reference group (BMI 18.5–24.9 kg/m^2^). There was a significantly lower rate of BV in post-menopausal obese women compared to the post-menopausal reference group, but not in pre-menopausal women. HIV- post-menopausal obese women had a significantly lower rate of BV, but this was not seen in HIV+ post-menopausal obese women. Pre-menopausal women with a higher hemoglobin A1c (≥6.5%) had a significantly lower rate (multivariable adjusted OR 0.66 (0.49–0.91), p = 0.010) of BV compared to pre-menopausal women with normal hemoglobin A1c levels (<5.7%), but there was no difference in post-menopausal women. This study shows an inverse association of BMI with BV in post-menopausal women and hemoglobin A1c with BV in pre-menopausal women. Further studies are needed to confirm these relationships in other cohorts across different reproductive stages and to identify underlying mechanisms for these observed associations.

## Introduction

The vaginal microbiota plays an important role in susceptibility to HIV and other sexually transmitted infections (i.e. *Neisseria gonorrhoeae*, *Chlamydia trachomatis* and others), pelvic inflammatory disease and premature delivery [1–3]. A vaginal microbiota that consists predominantly of bacteria in the genus *Lactobacillus* is associated with protection from these conditions and is characterized by a low vaginal pH from *Lactobacillus*-mediated lactic acid production. Conversely, bacterial vaginosis (BV), a condition where anaerobic non-*Lactobacillus* spp. bacteria dominate the vaginal microbiota, is associated with a higher vaginal pH and greater susceptibility to adverse outcomes. Several behaviors including sexual activity, smoking and vaginal douching affect the makeup of the vaginal microbiota [1, 4]. Menopause is also associated with decreased levels of vaginal *Lactobacillus* spp. [5, 6]. However, the main biological influences that prevent BV and lead to more beneficial vaginal bacteria types are poorly understood.

We previously found, among HIV-seronegative women enrolled in the Women’s Interagency HIV Study (WIHS), that levels of glycogen in the lumen of the vagina were higher in women that had a high BMI [7]. Glycogen released by the vaginal epithelium is thought to support growth of beneficial *Lactobacillus* spp. such as *L*. *crispatus* leading to decreased BV [7–10]. The conditions that influence the levels of vaginal glycogen are unknown, but in skeletal muscle, higher levels of blood glucose, as occurs with carbohydrate loading by athletes or in diabetes, have been associated with increased muscle glycogen [11, 12]. It is therefore plausible that increased blood glucose could also lead to increased vaginal glycogen levels which could be a mechanism for an association between BMI and increased genital glycogen. Since hemoglobin A1c (HbA1c) is routinely measured in the WIHS and reflects average blood glucose levels over the preceding 3 months [13], it could be measured to test the hypothesis that increased blood glucose impacts the genital microbiota.

The current study was undertaken to determine if BMI was associated with BV in women in the WIHS, an ongoing longitudinal cohort study of HIV-seropositive and demographically similar HIV-seronegative women initiated in 1994 across several US cities. Analyzing and comparing the effects of BMI on BV in both HIV+ and HIV- women is important since HIV infection and its treatments have effects on metabolism and body weight [14]. Additionally, HIV infection itself has been suggested to affect the makeup of the genital microbiota [15–18], with several studies finding an increased rate of BV in HIV+ women. Hemoglobin A1c values, available for a subset of the women, were also analyzed to determine if there was a significant relationship with BV. Elucidating the relationships between physiologic parameters and the vaginal microbiota could help identify ways to intervene to promote healthy vaginal microbiota, prevent BV and improve women’s reproductive health across reproductive stages.

## Materials and methods

The WIHS cohort consists of US women living with or at risk for HIV. Study methods have previously been described in detail [19–21]. Women in the cohort have twice-yearly visits/evaluations. For the purposes of this study, participant visits from October 1, 1994 through September 30, 2016 were included in this analysis. Participant visits in which the participant reported vaginal douching, use of vaginal medication, vaginal sex 48 hours before the visit, pregnancy, breastfeeding, and/or were less than 12 weeks postpartum were excluded from analyses because these factors potentially influence vaginal microbiota. In total, 10,184 person visits (15.3%) were excluded, consisting of; 1337 for douching in previous 48 hours; 1167 for vaginal meds use in previous 48 hours; 7584 for vaginal sex in previous 48 hours; 842 for current pregnancy; 230 for breastfeeding; and 166 for 12 weeks postpartum.

The parent WIHS study and this data analysis conformed to the procedures for informed written consent approved by institutional review boards (IRB) at all sponsoring organizations and to human-experimentation guidelines set forth by the United States Department of Health and Human Services, and finally reviewed and approved by the Cook County Health Review Board.

### Bacterial vaginosis

Bacterial vaginosis was identified by the presence of vaginal fluid pH >4.5 and at least 2 of the 3 other Amsel criteria: wet mount clue cells, amine odor of vaginal fluid when KOH is added (whiff test), and presence of a white/gray homogenous vaginal discharge. This is a modification of the standard Amsel criteria. However, for the first eight visits occurring from October 1, 1994 through September 30, 1998 bacterial vaginosis was identified as having all three of the following criteria; the presence of a vaginal fluid pH >4.5; wet mount clue cells; and amine odor of vaginal fluid when KOH is added. There were 10,047 pre-1998 visits included in this study.

### Body Mass Index (BMI)

BMI (kg/m^2^) was categorized as underweight (<18.5), normal (18.5–24.9), overweight (25.0–29.9) or obese (> = 30.0). Normal BMI was set as the reference group.

### Menopausal status

Person visits were retrospectively categorized using available data as menopausal (surgical or natural) if a woman reported bilateral oophorectomy or hysterectomy and/or no menses for >12 months with no subsequent resumption of menses during followup observations or pre-menopausal if a woman reported any menses within the past 6 months or subsequently resumed menses after a period of amenorrhea.

### Covariates

Covariates including potential confounders were selected a priori and based on previous literature and included age at visit, education level (<high school, completed high school, or >high school), alcohol use (>7 drinks per week or ≤7 drinks per week), tobacco use (current or none), drug use defined as use of crack, cocaine, and/or heroin (current or none), marijuana use (current or none), sexual activity and condom use (no vaginal sex since last visit, condom use always with one or more partners, sometimes or never condom use with one partner, or sometimes or never condom use with more than one partner), WIHS site (Bronx, Brooklyn, Washington D.C., Los Angeles, San Francisco, Chicago, Chapel Hill, Atlanta, Miami, Birmingham, or Jackson), parity (≥ one pregnancy or no pregnancies), recent yeast infection (self-reported infection since last visit or no recent infection), hormonal contraceptive use (current or none), absolute neutrophil count, hypertension (current vs previous or none), and chronic kidney disease (estimated glomerular filtration rate: ≥90, 60–89, 30–59, 15–29, and <15).

Race (black, other, white) and ethnicity (Hispanic, not Hispanic) were included as a composite variable (black, Hispanic, other, white), those that self-reported Hispanic ethnicity were categorized as Hispanic regardless of self-identified race.

HIV serostatus was included as a covariate and analyses were stratified by HIV status. For HIV+ only multivariable models, CD4 count (≥200, <200) was also included. All available HbA1c (≥6.5%, 5.7–6.4%, or <5.7%) measurements were also included in analysis (the WIHS measured this parameter in all women at a subset of visits). The frequency of genital infections was self-reported as being told by a healthcare provider that the participant had an infection since the last visit: gonorrhea 0.21%, syphilis 0.18%, chlamydia 0.43%, pelvic inflammatory disease 0.31%, Herpes 3.23%, trichomoniasis 1.44% and yeast 9.05%. Because yeast infection was relatively frequent, this was included in statistical modelling.

### Statistical analysis

Bivariate analyses using chi-square and Mann-Whitney tests assessed the relationship between BV at a visit and sociodemographic and clinical variables, by menopausal status and HIV serostatus. To account for repeated measures within participants, generalized estimating equation (GEE) adjusted odds ratios and 95% confidence intervals were obtained. Multiple multivariable (GEE) logistic regression models considered i) the association of BV and BMI (underweight, normal, overweight, and obese); ii) the association of BV and BMI stratified by menopausal status; iii) among the subset of participant visits with hemoglobin A1c data, the association of BV and BMI and BV and hemoglobin A1c, stratified by menopausal status; and iv) the previous model was also run separately for women living with HIV and HIV uninfected participants. The multivariable models adjusted for confounders including age at visit, HIV serostatus, race/ethnicity, education, alcohol use, cigarette use, drug use, marijuana use, sexual activity and condom use, parity, recent vaginal yeast infection, hormonal contraception, absolute neutrophil count, hypertension, and enrollment site. All analyses were performed using Statistical Analysis Software (SAS) software, version 9.4 (SAS Institute, Inc., Cary, NC).

## Results

We identified 4,637 women (3,451 HIV+ and 1,186 HIV-) assessed in WIHS for BV, contributing to 56,537 visits; BV was found at 6,770 (12.0%) of those visits. Among women living with HIV, BV was found at 5,435 visits (11.6%) and at 1,335 visits (13.7%) among HIV uninfected women. The average age (range) at time of enrollment of the women analyzed in this study was 37 years (range: 18–73). Other demographic, behavioral and physiologic data are shown in Table 1 for the index visit of the women in this study as well as the number of visits.

**Table 1:** Demographics of 4,637 women at their index visit and all person-visits included in this study.

	Overall at Index Visit (n = 4,637) N (%)	Overall (all person visits) (n = 56,537) N (%)
**Bacterial vaginosis**		
Positive	750 (16.2)	6,770 (12.0)
Negative	3,887 (83.8)	49,767 (88.0)
**Body mass index**		
Underweight, <18.5	125 (2.7)	1,454 (2.6)
Normal, 18.5–24.9	1,528 (33.0)	16,677 (29.5)
Overweight, 25.0–29.9	1,301 (28.0)	16,211 (28.7)
Obese, ≥30.0	1,683 (36.3)	22,195 (39.2)
**Age**, Mean (Range)	37 (18–73)	43 (18–83)
**HIV serostatus**		
Seropositive	3,451 (74.4)	46,775 (82.7)
Seronegative	1,186 (25.6)	9,762 (17.3)
**Menopausal status**^**b**^		
Pre-menopausal	3,802 (82.0)	33,200 (58.7)
Menopausal	835 (18.0)	23,337 (41.3)
**Race**		
African American	2,649 (57.1)	30,166 (53.4)
Other	1,240 (26.8)	17,659 (31.2)
White	748 (16.1)	8,712 (15.4)
**Ethnicity**		
Hispanic	1,024 (22.1)	13,899 (24.6)
Not Hispanic	3,613 (77.9)	42,638 (75.4)
**Education level**		
<HS	1,665 (35.9)	20,253 (35.8)
HS	1,409 (30.4)	17,277 (30.6)
>HS	1,563 (33.7)	19,007 (33.6)
**Drinks per week**, >7	627 (13.5)	5,340 (9.5)
**Current smoker**	2,378 (51.3)	25,223 (44.6)
**Current crack, cocaine, or heroin use**	1,034 (22.3)	6,019 (10.7)
**Current marijuana use**	1,140 (24.6)	9,813 (17.4)
**Sexual activity/condom use**		
No recent vaginal sex	1,303 (28.1)	23,522 (41.6)
Condom use always, ≥1 partner	1,530 (33.0)	18,796 (33.2)
Sometimes/never condom use, 1 partner	1,192 (25.7)	11,562 (20.5)
Sometimes/never condom use, >1 partner	612 (13.2)	2,657 (4.7)
**Parity**, ≥1	3,620 (78.1)	45,317 (80.2)
**Recent yeast infection**	1,240 (26.7)	5,628 (10.0)
**Hormonal contraceptive use**	519 (11.2)	3,858 (6.8)
**Absolute neutrophil count**, Median (IQR)	2,670 (1,822.5–3,731.8)	2,645 (1862.0–3690.0)
**Hypertension**, yes	1,392 (30.0)	19,829 (35.1)
**Geographic site**		
Bronx, NY	741 (16.0)	10,228 (18.1)
Brooklyn, NY	619 (13.3)	10,315 (18.2)
Washington DC	574 (12.4)	7,927 (14.0)
Los Angeles, CA	731 (15.8)	8,919 (15.8)
San Francisco, CA	629 (13.6)	8,591 (15.2)
Chicago, IL	526 (11.3)	7,871 (13.9)
Chapel Hill, NC	185 (4.0)	586 (1.0)
Atlanta, GA	267 (5.8)	838 (1.5)
Miami, FL	147 (3.2)	515 (0.9)
Birmingham, AL	109 (2.3)	380 (0.7)
Jackson, MS	109 (2.3)	367 (0.7)
***Subset of 3*,*543 participants at 16*,*306 visits with Hemoglobin A1c data***		
**Hemoglobin A1c**		
Normal, <5.7%	2,368 (66.9)	9,696 (59.5)
Pre-diabetes, 5.7–6.4%	890 (25.1)	5,044 (30.9)
Diabetes, ≥6.5%	285 (8.0)	1,566 (9.6)

Data presented as counts (frequencies), unless otherwise noted.

^a^Bacterial Vaginosis determined using Amsel Criteria. At least three out of four of the following positive: vaginal pH >4.5, characteristic discharge, KOH odor, and presentation clue cells.

^b^Menopausal status: menopausal–surgical menopause (hysterectomy or bilateral oophorectomy) and/or no menses in one year and no later resumption of menses. Pre-menopausal—menses within one year or prolonged amenorrhea with later resumption of menses.

The reference group (women with normal BMI, 18.5–24.9) had BV at 12.6% of visits. Adjusting for repeated measures, obese women (BMI >30) had a significantly lower likelihood of BV than the reference group (Table 2), even after adjusting for the covariates (OR 0.87 (0.79–0.97), p = 0.009). Overweight women (BMI 25.0–29.9) had lower rates of BV when compared to women with a normal BMI (18.5–24.9) in GEE univariable but not in multivariable adjusted analysis. Underweight women (BMI <18.5) did not have a different rate of BV than normal weight women.

**Table 2:** Relationship of BV with body mass index.

N (%)	BV at visit (n = 6,770)	No BV at visit (n = 49,767)	GEE Univariable OR (95% CI)	p value	Multivariable Adjusted OR (95% CI)	p value
**Body Mass Index**						
Underweight	235 (3.5)	1,219 (2.4)	1.03 (0.84–1.26)	.782	0.96 (0.79–1.17)	.707
Normal	2,108 (31.1)	14,569 (29.3)	Reference		Reference	
Overweight	1,886 (27.9)	14,325 (28.8)	**0.88 (0.81–0.96)**	**.004**	0.94 (0.86–1.04)	.218
Obese	2,541 (37.5)	19,654 (39.5)	**0.78 (0.70–0.86)**	**< .0001**	**0.87 (0.79–0.97)**	**.009**

Analysis adjusted for the following parameters; age at visit, menopausal status, HIV serostatus, race, education, alcohol, cigarettes, crack, cocaine, or heroin use, marijuana use, sexual activity/condom use, parity, recent vaginal yeast infection, hormonal contraception, absolute neutrophil count, hypertension, and WIHS enrollment site. Significant relationships are bolded.

We further examined whether the relationship between BMI and BV differed by menopausal status. In multivariable-adjusted analysis of post-menopausal women, the rate of BV was significantly lower in obese and overweight women than in women of normal weight (Table 3). However, among pre-menopausal women, there was no statistically significant difference in BV rate between obese women and normal weight women when adjusting for the covariates. Underweight women, whether pre- or post-menopausal, did not have significantly different rates of BV than the reference group in adjusted analyses.

**Table 3:** Relationship of BV with body mass index in pre- and post-menopausal women.

	Pre-Menopausal (n = 33,200)				Post-Menopausal (n = 23,337)			
	GEE Univariable OR	p value	Multivariable Adjusted OR	p value	GEE Univariable OR	p value	Multivariable Adjusted OR	p value
**Body Mass Index**								
Underweight	1.21 (0.96–1.54)	.111	1.04 (0.82–1.31)	.777	0.93 (0.69–1.27)	.655	0.87 (0.64–1.18)	.361
Normal	Reference		Reference		Reference		Reference	
Overweight	0.94 (0.85–1.04)	.205	1.00 (0.90–1.12)	.929	**0.83 (0.72–0.95)**	**.009**	**0.86 (0.73–0.99)**	**.047**
Obese	0.88 (0.79–0.99)*	**.029**	0.94 (0.84–1.06)	.330	**0.72 (0.62–0.83)**	**< .0001**	**0.77 (0.66–0.91)**	**.002**

*95% Confidence Interval, significant relationships are bolded.

Analysis adjusted for the following parameters; age at visit, HIV serostatus, race, education, alcohol, cigarettes, crack, cocaine, or heroin use, marijuana use, sexual activity/condom use, parity, recent vaginal yeast infection, hormonal contraception, absolute neutrophil count, hypertension, and WIHS enrollment site.

To investigate the hypothesis that increased blood glucose levels in women might be associated with a lower prevalence of BV, we examined available hemoglobin A1c levels. Hemoglobin A1c values were collected in all WIHS participants over a subset of visits, and were available for women at 16,306 of the WIHS visits (3,543 subjects: 2,615 HIV+, 928 HIV-). In this subgroup, obese women also had a lower rate of BV in multivariable adjusted analysis (not shown). Post-menopausal obese women had a significantly lower rate of BV compared to women with a normal BMI in adjusted analysis while pre-menopausal women did not (Table 4). In adjusted analysis of pre-menopausal women, those with higher hemoglobin A1c (≥6.5%) levels had a significantly lower rate (OR 0.66 (0.49–0.91), p = 0.01) of BV than women with normal levels of hemoglobin A1c (<5.7%), but this association was not found among post-menopausal women. (Table 4).

**Table 4:** Relationship between BV, BMI, and hemoglobin A1c in pre- and post-menopausal women.

	Pre-Menopausal (n = 8,950)				Post-Menopausal (n = 7,356)			
	GEE Univariable OR	p value	Multivariable Adjusted OR	p value	GEE Univariable OR	p value	Multivariable Adjusted OR	p value
**Body Mass Index**								
Underweight	1.75 (1.08–2.84)*	**.024**	1.54 (0.97–2.44)	.068	0.82 (0.48–1.40)	.467	0.74 (0.43–1.28)	.278
Normal	Reference		Reference		Reference		Reference	
Overweight	1.01 (0.85–1.21)	.885	1.07 (0.88–1.30)	.518	**0.74 (0.59–0.94)**	**.013**	0.78 (0.60–1.01)	.063
Obese	0.95 (0.79–1.14)	.582	0.93 (0.76–1.14)	.484	**0.71 (0.57–0.88)**	**.002**	**0.70 (0.54–0.89)**	**.005**
**Hemoglobin A1c**								
<5.7%	Reference		Reference		Reference		Reference	
5.7–6.4%	0.93 (0.80–1.08)	.365	0.87 (0.74–1.03)	.105	0.86 (0.72–1.02)	.085	0.87 (0.71–1.06)	.162
≥6.5%	**0.67 (0.50–0.91)**	**.011**	**0.66 (0.49–0.91)**	**.010**	**0.74 (0.56–0.98)**	**.038**	0.92 (0.65–1.28)	.604

*95% Confidence Interval, significant relationships are bolded.

Analyses adjusted for the following parameters; age at visit, HIV serostatus, race, education, alcohol, cigarettes, crack, cocaine, or heroin use, marijuana use, sexual activity/condom use, parity, recent vaginal yeast infection, hormonal contraception, absolute neutrophil count, hypertension, chronic kidney disease, and WIHS enrollment site.

In all of the analyses described above, the models controlled for HIV serostatus. We further examined the effect of HIV serostatus on the relationship between BMI, menopausal status, hemoglobin A1c, and BV. When stratified by menopausal status, post-menopausal obese women had a lower rate of BV in HIV seronegative, but not among HIV seropositive women when compared to normal weight women (Table 5). In multivariable-adjusted analyses, among pre-menopausal HIV seropositive women, those with poor glycemic control (hemoglobin A1c: ≥6.5%) also had significantly lower rates of BV than women with normal levels of hemoglobin A1c. This relationship was not seen in adjusted analyses among HIV-seronegative women.

**Table 5:** Relationship of BV with BMI and hemoglobin A1c in pre- and post-menopausal HIV-seropositive and -seronegative women.

	Pre-Menopausal				Post-Menopausal			
	GEE Univariable OR	p value	Multivariable Adjusted OR	p value	GEE Univariable OR	p value	Multivariable Adjusted OR	p value
***HIV positive (n = 13*,*155)***								
** Body Mass Index**								
Underweight	1.70 (0.98–2.96)	.060	1.50 (0.89–2.52)	.129	0.79 (0.43–1.47)	.457	0.63 (0.34–1.18)	.149
Normal	Reference		Reference		Reference		Reference	
Overweight	0.99 (0.81–1.21)	.922	1.04 (0.83–1.31)	.710	0.77 (0.59–1.01)	.059	0.81 (0.60–1.09)	.163
Obese	0.93 (0.75–1.14)	.477	0.92 (0.73–1.15)	.456	0.83 (0.64–1.06)	.134	0.80 (0.60–1.06)	.120
** Hemoglobin A1c**								
<5.7%	Reference		Reference		Reference		Reference	
5.7–6.4%	0.94 (0.79–1.12)	.503	0.89 (0.74–1.08)	.234	0.89 (0.73–1.08)	.236	0.92 (0.74–1.15)	.475
≥6.5%	**0.62 (0.42–0.90)**	**.013**	**0.65 (0.45–0.94)**	**.023**	0.76 (0.54–1.07)	.112	0.95 (0.65–1.40)	.813
***HIV negative (n = 3*,*151)***								
** Body Mass Index**								
Underweight	2.01 (0.80–5.07)	.138	1.73 (0.72–4.16)	.218	0.94 (0.34–2.60)	.906	0.88 (0.29–2.69)	.818
Normal	Reference		Reference		Reference		Reference	
Overweight	1.10 (0.74–1.65)	.629	1.16 (0.75–1.78)	.506	**0.61 (0.38–0.97)**	**.036**	0.66 (0.40–1.10)	.114
Obese	1.02 (0.70–1.50)	.910	0.98 (0.64–1.52)	.935	**0.35 (0.22–0.58)**	**< .0001**	**0.39 (0.23–0.66)**	**.0005**
** Hemoglobin A1c**								
<5.7%	Reference		Reference		Reference		Reference	
5.7–6.4%	0.87 (0.65–1.17)	.356	0.80 (0.57–1.12)	.197	0.70 (0.48–1.02)	.067	0.74 (0.49–1.12)	.156
≥6.5%	0.79 (0.48–1.30)	.357	0.70 (0.40–1.24)	.220	0.60 (0.34–1.06)	.078	0.74 (0.40–1.36)	.335

*95% Confidence Interval, significant relationships are bolded.

Analyses adjusted for the following parameters; age at visit, race, education, alcohol, cigarettes, crack, cocaine, or heroin use, marijuana use, sexual activity/condom use, parity, recent yeast infection, hormonal contraception, absolute neutrophil count, hypertension, chronic kidney disease, and WIHS enrollment site. HIV positive only models also adjusted for CD4 count.

## Discussion

This study found that, overall, participants in the WIHS that were obese (BMI>30) had a lower rate of BV than those with a normal BMI. This relationship between BMI and BV was only observed in post-menopausal women. HIV-seronegative post-menopausal obese women had a significantly lower rate of BV. Pre-menopausal, but not post-menopausal, women with elevated hemoglobin A1c had significantly lower BV than the reference group.

Several other studies assessed the relationship between BMI and BV with substantial differences from this study both in the characteristics of the population studied and the findings. However, none of those studies assessed the relationships between BMI and BV either in the context of menopause status or HIV status. Lokken et al. [22] studied a cohort of female sex workers in Mombasa Kenya and found that obese women had a lower rate of BV compared with the control group. One similarity between that study and the current WIHS study is that both cohorts included HIV-seropositive and HIV-seronegative women. However, there are several important differences in the studies. First, the racial and ethnic makeup of the two cohorts is substantially different. Second, the Mombasa study only assessed women 46 years old or younger (their stated proxy for menopause) while the WIHS cohort includes women that, over the years of the study, range in age from 18–83 yrs (the average age of post-menopausal women that had BV in the WIHS study was greater than 50 yrs). Additionally, menopause is assessed using menstrual cycle criteria in the WIHS cohort [23]. While it was not analyzed in the Lokken et al. study, it is possible that a portion of the significant inverse relationship they observed between BMI and BV could have been influenced by the age of women, with older obese women having lower rates of BV, as was found in this WIHS study. Another difference between the studies was that the Mombasa study used Nugent criteria for assessing BV for their main conclusions while this WIHS study used Amsel criteria for assessing BV. However, the Mombasa study also performed the Amsel in parallel to the Nugent and interestingly, the Amsel method also revealed that obese women had a lower rate of BV. The Mombasa study controlled for HIV serostatus during analysis but did not stratify or report whether HIV serostatus affected the relationship between BMI and BV.

Conversely, Brookheart et al. [24] found that obese women had a higher rate of BV when analyzing Nugent scores from 5,918 US women in the Contraceptive CHOICE Project. Our study did not observe a higher rate of BV in obese pre-menopausal women. A substantial difference from the current WIHS study was that recruitment for CHOICE targeted women ages 14–45 years old and mean age was 25.3 years old for all participants. Also, the CHOICE group were mainly HIV-seronegative. It is not clear why the CHOICE study found a higher rate of BV in obese women while the current WIHS study and the study by Lokken et al. did not observe this relationship in pre-menopausal women.

Kancheva et al. [25] found in a small study of copper IUDs in Thai women with a median age of 39, that those with a BMI <20 had a higher prevalence of BV. This WIHS study also observed a significantly higher rate of BV in pre-menopausal women with a BMI <18.5 (Table 4), although in adjusted analysis this was not significant. Mastrobattista et al. [26] evaluated whether BMI would affect the outcome of treatment of BV. However, that study was performed in pregnant women (excluded from our analysis), and no effect of BMI on treatment was observed. Given the many dramatic changes in physiology caused by pregnancy, it would not be surprising if pregnancy can affect any relationships between BMI and BV.

Previous studies provide evidence that vaginal glycogen can lead to growth of vaginal lactobacilli [7, 9, 10]. We did not measure vaginal glycogen levels in the women in this WIHS study. However, based on our previous study where we observed that obese women had higher levels of vaginal glycogen [7], we predicted that obese women might have a lower rate of BV. Further, since ingestion of higher levels of carbohydrates and higher levels of blood glucose are associated with higher muscle glycogen [11, 12], we predicted that there would be a significant negative relationship between BV and hemoglobin A1c. However, while obese post-menopausal women were less likely to have BV, post-menopausal women with elevated hemoglobin A1c did not have a lower rate of BV suggesting that in obese post-menopausal women, the lower BV levels were not caused by increased glycogen due to higher glucose. Interestingly, pre-menopausal women with elevated hemoglobin A1c had significantly lower BV than the reference group of hemoglobin A1c <5.7% (Table 4) possibly indicating that increased blood glucose had an effect through glycogen although this apparently was not related to BMI. To our knowledge there are no studies of the effect of chronic diabetes on the rate of BV. Two studies examined women with gestational diabetes mellitus (GDM) and found no difference in the rate of BV from controls with no GDM [27, 28]. African American women have been observed to have generally higher levels of diabetes and obesity and it will therefore be important for further studies to determine if ethnic or racial backgrounds affect the relationship between diabetes, obesity and BV.

This study provides evidence that two very common conditions, obesity and diabetes, can affect the vaginal microbiota. The rate of obesity in women worldwide can be as high as 10–15%, and in the US, the rate is much higher [29]. Within some HIV-infected cohorts, aging is associated with increased obesity [30]. Additionally, HIV-infected African Americans have higher rates of obesity [31]. The prevalence of diabetes is approximately 10% in the US [32]. Diabetes is highly prevalent in HIV-infected persons (both men and women) [30]. Our current study found that while HIV- post-menopausal obese women had a significantly lower rate of BV, this relationship was not seen in HIV+ post-menopausal obese women. The immune system is significantly impacted during HIV infection which could affect the relationship between obesity and BV and may help explain this difference. There are few if any studies that address the impact of HIV infection on BV in post-menopausal women. However, several studies found that HIV infection is associated with changes in the vaginal microbiota in pre-menopausal women [15, 16]. In contrast, Massad et al. [18] found that hygienic and sexual practices such as sexual practices and smoking affected development of BV while HIV infection itself had little impact on BV rates.

While this study found an association between obesity and the rate of BV, this relationship did not appear to be due to increased blood glucose as measured by hemoglobin A1c. Our previous study showed however, that vaginal glycogen was increased in obese women and it is therefore possible that the lower rate of BV we observed in obese women in the WIHS was due to higher levels of vaginal glycogen. While estrogen has been posited to affect vaginal glycogen, in a previous study we did not find any association between vaginal glycogen and estrogen [10]. A separate study in menopausal women also did not find a significant relationship between vaginal glycogen and serum estrogens [33]. However, the relationship between glycogen, obesity and estrogen are highly complex. For example, in menopausal women, estrogen replacement therapy impacts body fat distribution and may reduce obesity [34]. Also, ovariectomy in animal models can lead to increased body weight [35].

Therefore, further research is needed to determine the underlying causes of the association between obesity, increased hemoglobin A1c and bacterial vaginosis.

There were several limitations to this study. We found early onset menopause (age <50 years of age, prolonged amenorrhea, and no later resumption of menses) in 1,874 (3.32%) of participant visits. At the index visit and using this definition, 18% of women were in menopause and by the end of the study 33.2% of participants were menopausal. Based on our definition of menopause in this study, we are possibly mislabeling a small subset of women as menopausal who have prolonged, but eventual reversible amenorrhea just not captured. It is also unclear whether these women with no later resumption of menses are all truly post-menopausal, or if there is another or unknown etiology for the prolonged amenorrhea. Additionally, some data were excluded (see methods) and this could have biased results. Further, this study determined BV using the Amsel criteria instead of Nugent’s Gram stain criteria [36], which has been suggested as more rigorous in the identification of BV. The Amsel criteria is considered to be highly specific, with most of found cases likely true cases. However, Amsel is not highly sensitive and it is possible that cases of BV were missed; rates of BV were higher in a previous single site WHIS study where BV rates based on Nugent scoring were 33% [37]. Additionally, there was a change in the Amsel criteria used to define BV in 1998 which may have impacted some of the findings. Interestingly, in a cross-sectional analysis of a single visit of data from the WIHS where data for both tests was available, BV by Amsel Criteria and BV by Nugent score (7 out of 10) were strongly associated (odds ratio = 45.7, 95% confidence interval: 21.4, 97.3) (ED unpublished observation) (Nugent data was not collected for all WIHS visits).

Despite these weaknesses, there were also several strengths to the study. The WIHS is a large longitudinal cohort study conducted over several decades, so there is a relatively large amount of data available spanning several reproductive stages. Also, the WIHS has sites throughout the contiguous US providing geographic representation, as well as the ability to control for areas with higher rates of STI acquisition.

In summary, in the WIHS cohort, obese post-menopausal women had a significantly lower BV rate compared to post-menopausal women with a normal BMI. To our knowledge, this is the first study to report the relationship between BV and BMI in post-menopausal women. This relationship of BMI and BV was not seen in pre-menopausal women and the relationship did not appear to be related to blood glucose levels in post-menopausal women. However, in pre-menopausal women with a higher hemoglobin A1c (≥6.5%), there was a significantly lower BV rate. These results suggest that the role of glycemia, vaginal glycogen in pre- and post-menopausal women, and the relationship with BV should be further explored.
